# Implications for SARS-CoV-2 Vaccine Design: Fusion of Spike Glycoprotein Transmembrane Domain to Receptor-Binding Domain Induces Trimerization

**DOI:** 10.3390/membranes10090215

**Published:** 2020-08-30

**Authors:** Taha Azad, Ragunath Singaravelu, Mathieu J.F. Crupi, Taylor Jamieson, Jaahnavi Dave, Emily E.F. Brown, Reza Rezaei, Zaid Taha, Stephen Boulton, Nikolas T. Martin, Abera Surendran, Joanna Poutou, Mina Ghahremani, Kazem Nouri, Jack T. Whelan, Jessie Duong, Sarah Tucker, Jean-Simon Diallo, John C. Bell, Carolina S. Ilkow

**Affiliations:** 1 Ottawa Hospital Research Institute, Ottawa, ON K1H 8L6, Canada; tazad@ohri.ca (T.A.); rsingaravelu@ohri.ca (R.S.); mcrupi@ohri.ca (M.J.F.C.); tjamieson@ohri.ca (T.J.); jdave@ohri.ca (J.D.); emibrown@ohri.ca (E.E.F.B.); Reza.Rezaei@uottawa.ca (R.R.); ztaha@ohri.ca (Z.T.); sboulton@ohri.ca (S.B.); nikmartin@ohri.ca (N.T.M.); asure077@uottawa.ca (A.S.); ypoutoupaumier@ohri.ca (J.P.); jwhelan@ohri.ca (J.T.W.); jduong@ohri.ca (J.D.); satucker@ohri.ca (S.T.); jsdiallo@gmail.com (J.-S.D.); jbell@ohri.ca (J.C.B.); 2 Department of Biochemistry, Microbiology and Immunology, University of Ottawa, Ottawa, ON K1H 8M5, Canada; 3 Department of Biology, University of Ottawa, Ottawa, ON K1N 6N5, Canada; mghahre2@uottawa.ca; 4 Princess Margaret Cancer Centre, University Health Network, Toronto, ON M5G 2C1, Canada; kazem.nouri@gmail.com

**Keywords:** trimerization, spike protein, receptor-binding domain, transmembrane domain, SARS-CoV-2, vaccine development

## Abstract

The ongoing severe acute respiratory syndrome coronavirus 2 (SARS-CoV-2) pandemic presents an urgent need for an effective vaccine. Molecular characterization of SARS-CoV-2 is critical to the development of effective vaccine and therapeutic strategies. In the present study, we show that the fusion of the SARS-CoV-2 spike protein receptor-binding domain to its transmembrane domain is sufficient to mediate trimerization. Our findings may have implications for vaccine development and therapeutic drug design strategies targeting spike trimerization. As global efforts for developing SARS-CoV-2 vaccines are rapidly underway, we believe this observation is an important consideration for identifying crucial epitopes of SARS-CoV-2.

## 1. Introduction

As the current severe acute respiratory syndrome coronavirus 2 (SARS-CoV-2) pandemic continues, there is a pressing need for the development of effective vaccine candidates. According to the World Health Organization, as of the end of August 2020, there are more than 30 candidate vaccines in clinical evaluation and 140 candidate vaccines in preclinical studies [1]. SARS-CoV-2 receptor-binding and entry is initiated upon the interaction of the protruding virion-associated spike (S) glycoprotein with the viral receptor, angiotensin-converting enzyme 2 (ACE2) on target cells, primarily in the lower respiratory system [2].

Studies have shown that the receptor-binding domain (RBD) of the spike protein is highly specific and is an immunodominant target for neutralizing antibodies [3,4]. Many candidate vaccines currently in clinical development have incorporated RBD as the target antigen. Further, it has been established that the spike protein ectodomain results in trimerization, and this has paved the way for vaccine development efforts targeting the trimeric spike protein [5,6,7]. However, studies to date have not yet investigated the role of the SARS-CoV-2 spike protein transmembrane domain (TMD) in protein trimerization. While previous work has demonstrated that the TMD of SARS-CoV, the etiologic agent responsible for the 2002-2003 SARS epidemic, is highly conserved [8], and important for membrane fusion and cell-cell fusion activity [8,9], it remains unclear whether the SARS-CoV-2 TMD plays any role in trimerization.

Interestingly, Tai et al. showed that the recombinant receptor-binding domain of MERS-CoV when expressed in a trimeric form induced a potent neutralizing antibody response in vivo and protected transgenic mice from MERS-CoV infection [10]. Thus, determining analogous strategies to induce SARS-COV-2 spike RBD trimerization may have a notable impact on vaccine development, especially since more than 15 vaccine platforms, currently in development, are based on the RBD. Herein, we demonstrate that SARS-CoV-2 TMD induces trimerization of the spike RBD, which may be an important consideration for platforms that are making vaccines using the RBD as an immunogen.

## 2. Materials and Methods

### 2.1. Cell Culture

HEK293T (CRL-3216), Vero (CCL-81), and U-2 OS (HTB-96) cell lines were obtained from the American Type Culture Collections (Manassus, VA, USA). Cells were maintained in Dulbecco’s modified Eagle’s medium (DMEM) (Thermo Fisher Scientific, Waltham, MA, USA) supplemented with 10% fetal bovine serum (FBS) (Thermo Fisher Scientific, Cat.#SH30396.03) and 1% penicillin–streptomycin (Invitrogen, Carlsbad, CA, USA).

### 2.2. Plasmid Construction

Inserts (See Supplemental Table S1 for detailed sequences) were ordered from GenScript (Piscataway, NJ, USA). SARS-CoV-2 RBD or RBD-TMD constructs were cloned into the BamHI/NotI sites of pcDNA3.1 or the XhoI/NheI sites of vesicular stomatitis virus (VSV) backbone plasmid. The DAN and amino acid sequences are available in Figure S1.

### 2.3. Virus Rescue

To rescue the recombinant VSV viruses expressing SARS-CoV-2 RBD or RBD-TMD, HEK293T cells were infected with vaccinia virus expressing T7 RNA polymerase (MOI = 3) for 2 h. Inoculum was removed and cells were transfected with the viral backbone plasmid along with T7 RNA polymerase-driven expression plasmids for VSV N, L, and P genes. Forty-eight hours post-transfection, the recombinant virus was collected, filtered (0.2 um) and plaque purified in Vero cells.

### 2.4. SDS-PAGE Gel Electrophoresis

Whole cell lysates were obtained by lysing the HEK293T cells in RIPA buffer (pH 7.4 Tris-HCl 25mM, NaCl 150mM, NP-40 1%, sodium deoxycholate 0.5%, SDS), and 1× protease inhibitor cocktail (Roche, Basel, Switzerland) on ice. Protein concentration was determined by Pierce bicinchoninic acid (BCA) assay (Thermo Scientific, Cat.# 23225) and 10µg of cell extract were mixed into DTT-Laemmli buffer and boiled for 5 min. Samples were resolved using the NuPAGE SDS-PAGE system (Invitrogen, Carlsbad, CA, USA, Cat. # NP0322) for 1.5 h at constant voltage (150 V), then transferred onto a nitrocellulose membrane (BioRad, Cat. # 1620115).

### 2.5. Native-PAGE Gel Electrophoresis 

Whole cell lysates were harvested from recombinant VSV infected HEK293T cells using NativePAGE sample kit (Invitrogen, Cat.# BN2008). Samples were homogenized in buffer containing 2% digitonin on ice and centrifuged to clarify the lysates. Collected lysates were boiled at 100 °C for 5 min, then 10 µg of total proteins were loaded onto a pre-cast NativePAGE Bis-Tris gel (Invitrogen, Cat.# BN1002BOX).

The NativePAGE Novex Bis-Tris gel system was used with an XCell SureLock Mini-Cell gel tank (Invitrogen). The upper cathode chamber was filled with 200mL of 1X NativePAGE dark blue cathode buffer (BN2002), and the lower anode chamber was filled with 550mL of NativePAGE anode (running) buffer (Invitrogen, BN2001). Once the dye front reached 1/3 the length of the gel, the dark blue cathode buffer was replaced with light blue cathode buffer. The gel was run for 1.5 h at a constant voltage of 150V. 

### 2.6. Immunoblotting of PAGE Gels 

Following gel electrophoresis, proteins were transferred to Immobilon-P polyvinylidene fluoride (PVDF) membrane (MilliporeSigma, Burlington, MA, USA) overnight at 4 °C. The PVDF membrane was blocked for 1 h in 5% milk in Tris-buffered saline with 0.1% Tween 20 (TBS-T), washed in TBS-T, then probed for 1 h at room temperature with mouse anti-FLAG (1:1000, MilliporeSigma, Cat.#F3165). Blots were then washed and incubated with anti-mouse (1:5000, MilliporeSigma, Cat.#A9044) for 1 h at room temperature. Blots were imaged using the ChemiDoc MP imaging system (Bio-Rad Laboratories, Mississauga, ON, USA). SuperSignal West Pico PLUS Chemiluminescent Substrate (Thermo Fisher Scientific, Cat. #34577) was used to visualize the protein bands.

### 2.7. Dot Blot Assay

U-2 OS cells, infected with VSV viruses at an MOI of 0.1, were harvested 24 h post-infection in RIPA buffer supplemented with 1× protease/phosphatase inhibitor cocktail (Cell Signaling Technology, Danvers, MA, USA, Cat. #5872S) on ice. Protein concentration was determined by BCA assay, and 10 µg of whole cell lysates was directly loaded onto nitrocellulose membrane (Bio-Rad Laboratories) with 1 h incubation at room temperature. After blocking in 5% milk in TBS-T, membranes were probed with either mouse anti-RBD clone 5B7D7 (GenScript, Cat.#A02056), clone 6D11F2 (GenScript, Cat.#A02055), or clone 40592 (Sino Biological, Wayne, PA, USA); mouse anti-VSV-G clone 8G5F11 (Kerafast, Boston, MA, Cat.#EB0010), or rabbit anti-β-actin clone 13E5 (Cell Signaling Technology, Cat.#4970S) as a loading control; ectodomain ACE2 and goat anti-ACE2 (R&D Systems, Minneapolis, MN, USA, Cat.#AF933), or sera from BALB/cJ mice (The Jackson Laboratory, Bar Harbor, ME, USA) immunized with SARS-CoV-2 spike. Blots were then washed and probed with appropriate secondary antibodies: anti-mouse, anti-rabbit (MilliporeSigma, Cat. #A9169) or anti-goat (Abcam, Cambridge, UK, Cat.#ab97110). Blots were imaged using the ChemiDoc MP imaging system (Bio-Rad Laboratories, Mississauga, ON, USA). Clarity Western ECL Substrate (Bio-Rad) was used to visualize the blot.

### 2.8. Conservation and Phylogenetic Analysis

A total of 10,318 surface glycoprotein sequences of SARS-CoV-2 were retrieved from the particular NCBI dataset page for this virus. These sequences were aligned using ClustalOmega software [11]. Jalview software was used for visualization and conservation score calculations [12]. For inter-species phylogenetic analysis, the target sequence was queried in NCBI protein blast [13,14] against the Betacoronavirus genus (txid:694002). After removing redundant identical sequences, the set of sequences were selected for tree construction and multiple sequence alignment generation. All steps were run using default settings except the use of the neighbor-joining algorithm for phylogenetic tree calculation in the NCBI website.

## 3. Results and Discussion

**Figure 1:**
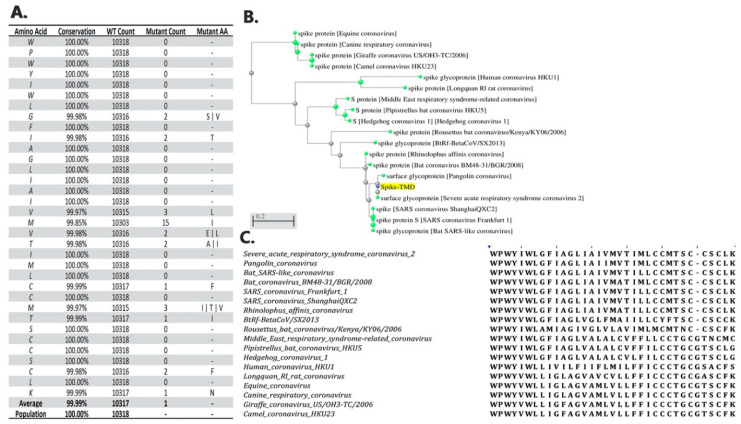
Intra-species conservation and inter-species phylogenetic analyses of the spike transmembrane domain. (**A**) Conservation score of the spike transmembrane domain (TMD) protein sequence between all submitted sequences for surface glycoprotein in severe acute respiratory syndrome coronavirus 2 (SARS-CoV-2) samples. (**B**) Phylogenetic tree of the spike TMD sequence between different members of the Betacoronavirus genus. (**C**) the multiple sequence alignment visualization of the phylogenetic tree sequences.

**Figure 2:**
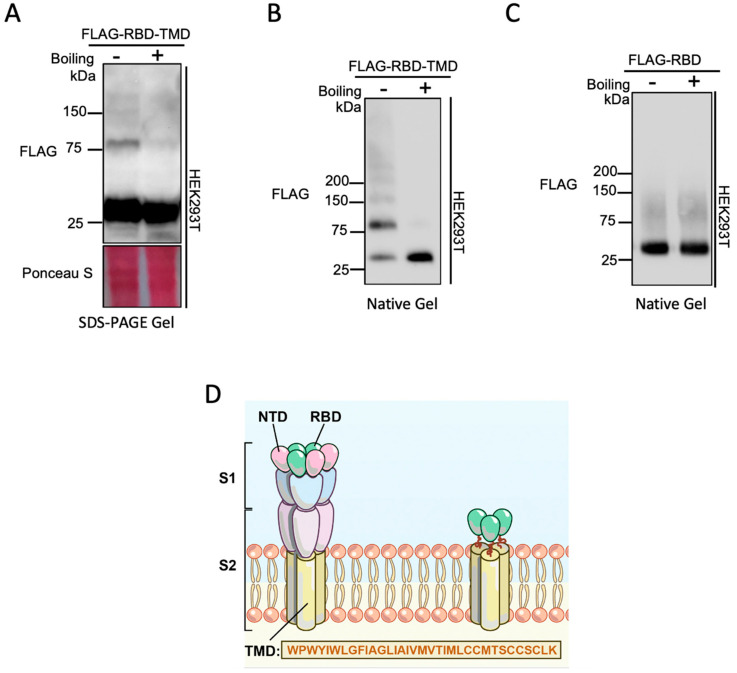
Characterizing the role of the transmembrane domain in the trimerization of the receptor-binding domain. (**A**) SDS-PAGE immunoblot analysis of chimeric RBD with or without boiling the lysate prior to loading. Expected size of RBD is 25kDa. (**B**) Native-PAGE immunoblot of RBD alone with or without boiling the lysate prior to loading. (**C**) Native-PAGE gel analysis of chimeric RBD in the absence or presence of boiling sample prior to loading. (**D**) Schematic of SARS-CoV-2 spike glycoprotein and the chimeric RBD linked to S-TMD. N-terminal domain (NTD); receptor-binding domain (RBD); transmembrane domain (TMD).

A close examination of the 10,318 available SARS-CoV-2 genome sequences from NCBI datasets reveals more than 99.99% conservation of the TMD amino acid sequence (Figure 1). The high conservation of the TMD sequence between Betacoronaviruses suggests an important role for this domain in coronavirus infection. To examine the contribution of the TMD to spike trimer formation, a chimeric construct was made by fusing the TMD to the RBD (henceforth referred to as RBD-TMD). This construct was expressed in HEK293T cells and cell lysates were resolved using SDS-PAGE. Interestingly, in the absence of protein denaturation heat treatment, we observed an expected robust band at 25 kDa that corresponds to the monomeric chimeric RBD construct, but also a 75 kDa band, as well as the presence of a faint, high molecular weight band at 150 kDa (Figure 2A). We postulated that these higher molecular weight bands are the result of trimerization (75 kDa) or oligomerization (150kDa) of RBD-TMD.

We next investigated the effect of thermal denaturation on the occurrence of the 75 kDa band. Heat treatment of the sample eliminated the 75 kDa band in the SDS-PAGE gel (Figure 2A). The RBD-TMD was also analyzed by native PAGE with and without thermal denaturation to explore the role of proper protein folding on the occurrence of the 75 kDa band. Under native conditions the relative intensity of the 75 kDa band was increased in comparison to the monomeric band at 25 kDa, and, similarly to the SDS-PAGE analysis, the larger band disappeared with heat treatment (Figure 2B). To determine whether it was the RBD or the TMD that was responsible for the occurrence of the 75 kDa band, the native PAGE analysis was repeated using the RBD alone (Figure 2C). Without the TMD, the presence of the 75 kDa band was largely diminished. This indicates that the larger band is not due to an interaction between the RBD and another protein, and that the RBD, by itself, lacks the capacity to facilitate trimerization.

**Figure 3:**
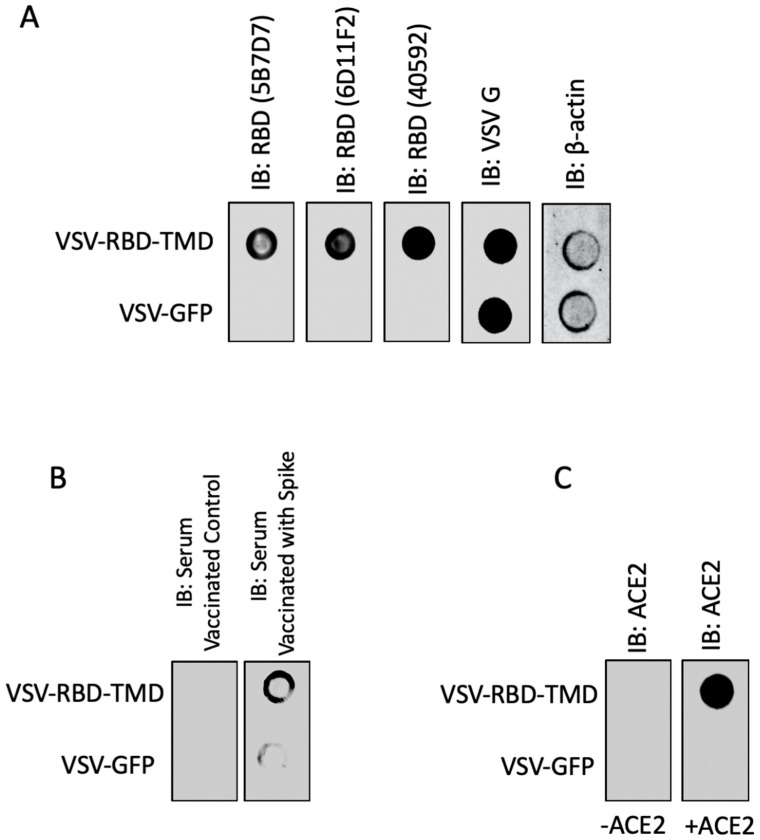
RBD-TMD fusion protein retains antigenic and functional conformation. U-2 OS cells were infected with vesicular stomatitis virus (VSV) expressing either GFP or RBD-TMD (MOI = 0.1). Twenty-four hours post-infection, dot blot analysis were performed, and membranes were probed using (**A**) RBD antibodies, (**B**) sera from mice immunized with control or SARS-CoV-2 spike, or (**C**) a combination of recombinant angiotensin-converting enzyme 2 (ACE2) protein and ACE2 antibody. VSV-G and β-actin indicate equal virus and loading levels, respectively.

Next, we sought to investigate whether the RBD-TMD trimerized protein complex retains RBD’s antigenic and functional conformation. Therefore, we constructed an attenuated vesicular stomatitis virus (VSV) expressing RBD-TMD to evaluate the conformation of the construct as a potential antigen encoded in a vaccine vector. Dot blot analysis revealed that both neutralizing antibodies targeting conformational epitopes in the RBD (Figure 3A) and mouse sera targeting spike (Figure 3B) recognized the RBD-TMD construct. These data suggest that the RBD-TMD trimer retains the antigenic conformation of the RBD. In addition, we also examined the capacity of RBD-TMD to interact with ACE2 using dot blot analysis (Figure 3C). Our results demonstrate that RBD-TMD binds ACE2—pointing to the fusion protein’s maintained capacity to interact with the SARS-CoV-2 host cell receptor. Collectively, our data demonstrate that the TMD fusion construct mediates trimerization of RBD while maintaining a relevant antigenic conformation for vaccine design.

The physiological and biological aspects of a TMD are important to explore, as both can affect receptor compartmentalization, membrane anchoring, and protein interactions. However, in the context of SARS-CoV-2, the role of the spike TMD in trimerization appears to have been overlooked. Although the ectodomain of spike occupies the majority of functions involved in viral entry and spread, we identify the TMD’s capacity to mediate RBD trimerization independently. Indeed, this highlights the important implications for RBD immunogenicity, specifically in the context of the therapeutic strategies aimed at the generation of neutralizing antibodies, as well as anti-trimerization approaches.

Many of the known vaccine platforms currently in development target the RBD of spike. Given our findings, we speculate that developing a vaccine which targets a chimeric RBD-TMD may be more effective in inducing protective immunity rather than targeting RBD alone. This finding is corroborated by past coronavirus vaccine initiatives, such as in the case of a MERS-CoV vaccine study. In that study, a chimeric RBD fused to a foldon trimerization motif elicited stronger neutralizing antibody responses and better lethal challenge protection than the comparable RBD-only vaccines [10].

## 4. Conclusions

Our data indicate that the SARS-CoV-2 virus TMD induces trimerization of RBD and emphasize the importance of investigating a chimeric RBD that includes the TMD of SARS-CoV-2 as a potential immunogen for vaccine development, or in anti-viral strategies targeting the RBD in SARS-CoV-2 infection. However, considering the hidden nature of the spike TMD and therefore the unlikelihood of it operating as a robust immunogen, we speculate that the employment of specific allosteric modulators of the TMD may be ideal. These allosteric modulators could include antibodies, recombinant antibody derivatives, or small molecule compounds that would bind to the ectodomain of spike and induce a conformational change that would render the TMD incapable of trimerization [15,16,17].

Given that many vaccines currently in development target RBD, we believe that it is necessary to have a holistic understanding of the mechanisms underlying the virulence capacity of the SARS-CoV-2 spike glycoprotein. Knowing that the TMD plays a critical role in the trimerization of spike preceding viral entry may now prompt new research streams aiming to tease apart the intricacies of these mechanisms in an effort to therapeutically target them. Coupling the targeting of trimerization with current anti-RBD strategies may yield a synergistic anti-viral strategy.

## Supplementary Materials

The following are available online at https://www.mdpi.com/2077-0375/10/9/215/s1. Figure S1. Uncropped blots. Table S1. Insert DNA and Amino acid sequence.
