# Supplementary material for: Implications for SARS-CoV-2 Vaccine Design: Fusion of Spike Glycoprotein Transmembrane Domain to Receptor-Binding Domain Induces Trimerization
Source: Membranes (Basel). 2020 Aug 30;10(9):215. doi: 10.3390/membranes10090215 (PMC7557813; doi:10.3390/membranes10090215)
Supplement: Supplementary file 1 [file membranes-10-00215-s001.pdf]

membranes

Supplementary Materials: Implications for SARS-CoV-2 Vaccine Design: Fusion of Spike Glycoprotein Transmembrane Domain to Receptor Binding Domain Induces Trimerization

Taha Azad [1,2,†], Ragunath Singaravelu [1,2,†], Mathieu J.F. Crupi [1,2,†], Taylor Jamieson [1,2,†], Jaahnavi Dave [1,2], Emily E.F. Brown [1,2], Reza Rezaei [1,2], Zaid Taha [1,2], Stephen Boulton [1,2], Nikolas T. Martin [1,2], Abera Surendran[1,2], Joanna Poutou[1,2], Mina Ghahremani[3], Kazem Nouri [4], Jack T. Whelan [1,2], Jessie Duong[1,2], Sarah Tucker [1,2], Jean-Simon Diallo [1,2], John C. Bell [1,2] and Carolina S. Ilkow [1,2,*]

1 Ottawa Hospital Research Institute, Ottawa, ON, K1H 8L6 and Canada.
2 Department of Biochemistry, Microbiology and Immunology, University of Ottawa, Ottawa, ON K1H 8M5, Canada.
3 Department of Biology, University of Ottawa, Ottawa, ON, K1N 6N5, Canada.
4 Princess Margaret Cancer Centre, University Health Network, Toronto, ON, Canada
* Correspondence: cilkow@ohri.ca; Tel.: +1-613-737-8899 (ext. 75208).
† These authors contributed equally.

Received: 11 August 2020; Accepted: 28 August 2020; Published: date

Uncropped blots

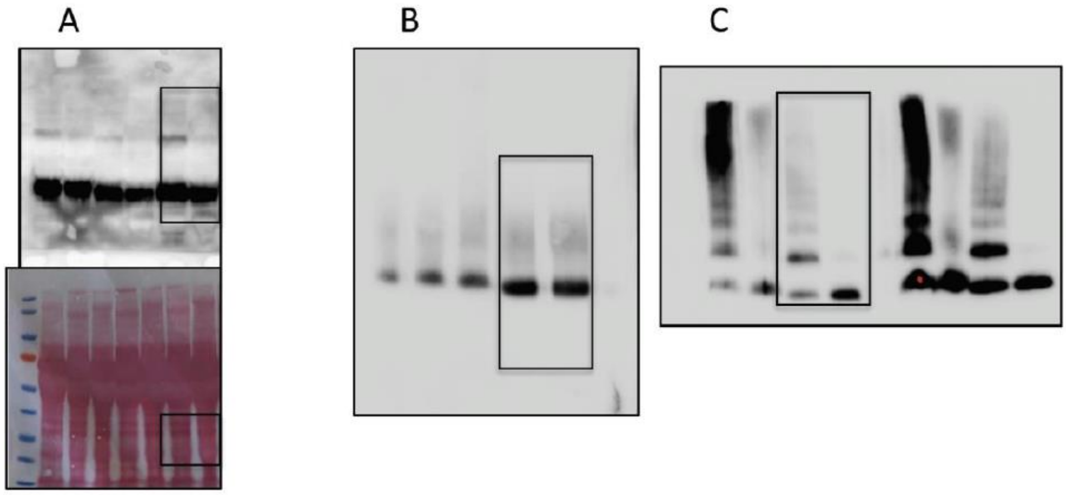

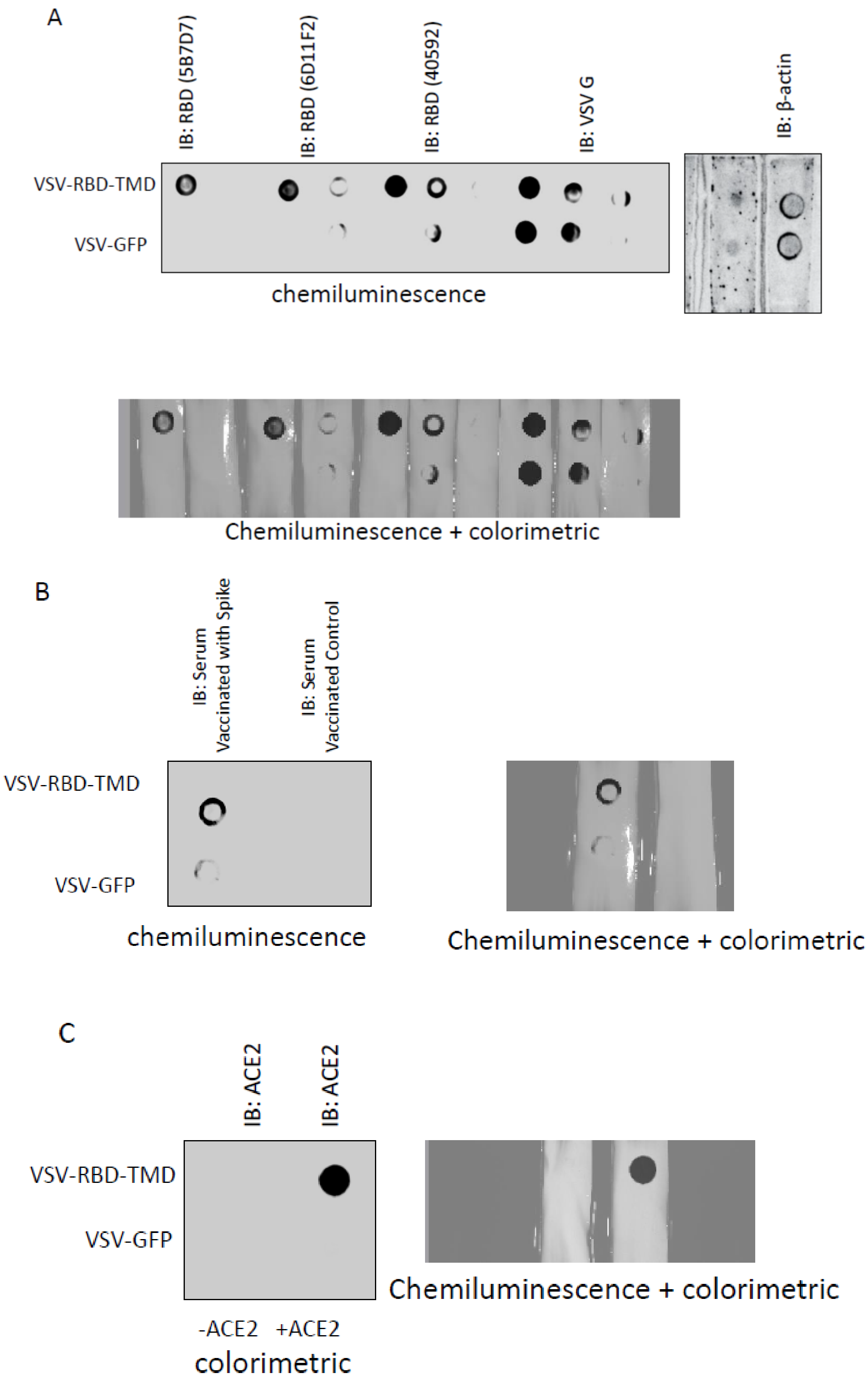

Figure S1. Uncropped blots.

Table S1. Insert DNA and Amino acid sequence.

Insert	DNA Sequence	Amino Acid Sequence
IgK-RBD-FLAG	ATGGAGACAGACACACTCCTGCTATGGGTACTGCTGCTCTGGG<br>TTCCAGGTTCCACTGGTGACTCTGGCTCTAGCGGCTCTGGCTCT<br>AGCGGCGGCATGGTGAGCGGCTGGCGGCTGTTCAAGAAGATT<br>AGCTCTAGCGGCGACTACAAGGACCACGACGGTGACTACAAG<br>GACCACGACATCGACTACAAGGACGACGACGACAAGGGCAG<br>CGGCTCCGGCAGCAGCGGAGGAGGAGGCTCTGGAGGAGGAG<br>GCTCTAGCGGCGGCAACATCACAAATCTGTGCCCATTCGGCGA<br>GGTGTTTAACGCCACCAGATTTGCCAGCGTGTATGCCTGGAAC<br>CGGAAGAGAATCTCTAATTGCGTGGCCGACTATAGCGTGCTGT<br>ACAATAGCGCCTCCTTCTCTACCTTTAAGTGCTATGGCGTGTCC<br>CCCACAAAGCTGAACGACCTGTGCTTCACCAACGTGTACGCCG<br>ACTCTTTTGTGATCAGGGGCGATGAGGTGCGCCAGATCGCACC<br>TGGACAGACAGGCAAGATCGCCGACTACAACTATAAGCTGCC<br>AGACGATTTCACCGGCTGCGTGATCGCCTGGAATAGCAACAAT<br>CTGGATTCCAAAGTGGGCGGCAACTACAATTATCTGTACCGGC<br>TGTTCAGAAAGAGCAACCTGAAGCCCTTTGAGCGGGATATCAG<br>CACAGAGATCTACCAGGCAGGCTCCACCCCTTGCAACGGAGT<br>GGAGGGCTTCAATTGTTATTTCCCCTGCAGAGCTACGGCTTCC<br>AGCCTACAAATGGCGTGGGCTATCAGCCATACAGGGTGGTGGT<br>GCTGTCCTTTGAGCTGCTGCACGCACCTGCAACCGTGTCCTCTG<br>GACACATCGAGGGCCGCCACATGCTGGAGATGGGCCATCATC<br>ACCATCATCACCACCACCACCACTGA	METDTLLLWVLLLWV<br>PGSTGDSGSSGSGSSG<br>GMVSGWRLFKKISSSG<br>DYKDHDGDYKDHDI<br>DYKDDDDKGSGSGSS<br>GGGGSGGGGSSGGNI<br>TNLCPFGEVFNATRF<br>ASVYAWNRKRISNCV<br>ADYSVLYNSASFSTFK<br>CYGVSPTKLNDLCFT<br>NVYADSFVIRGDEVR<br>QIAPGQTGKIADYNY<br>KLPDDFTGCVIAWNS<br>NNLDSKVGGNYNYL<br>YRLFRKSNLKPFERDIS<br>TEIYQAGSTPCNGVEG<br>FNCYFPLQSYGFQPTN<br>GVGYQPYRVVVLSFE<br>LLHAPATVSSGHIEGR<br>HMLEMGHHHHHHH<br>HHH
IgK-RBD-TMD-FLAG	ATGGAGACAGACACACTCCTGCTATGGGTACTGCTGCTCTGGG<br>TTCCAGGTTCCACTGGTGACTCTGGCTCTAGCGGCTCTGGCAGC<br>GGCGACTACAAGGACCACGACGGTGACTACAAGGACCACGAC<br>ATCGACTACAAGGACGACGACGACAAGGGAGGAGGAGGCTCT<br>AGCGGCGGCAACATCACAAATCTGTGCCCATTCGGCGAGGTGT<br>TTAACGCCACCAGATTTGCCAGCGTGTATGCCTGGAACCGGAA<br>GAGAATCTCTAATTGCGTGGCCGACTATAGCGTGCTGTACAAT<br>AGCGCCTCCTTCTCTACCTTTAAGTGCTATGGCGTGTCCCCCAC<br>AAAGCTGAACGACCTGTGCTTCACCAACGTGTACGCCGACTCT<br>TTTGTGATCAGGGGCGATGAGGTGCGCCAGATCGCACCTGGAC<br>AGACAGGCAAGATCGCCGACTACAACTATAAGCTGCCAGACG<br>ATTTCACCGGCTGCGTGATCGCCTGGAATAGCAACAATCTGGA<br>TTCCAAAGTGGGCGGCAACTACAATTATCTGTACCGGCTGTTC<br>AGAAAGAGCAACCTGAAGCCCTTTGAGCGGGATATCAGCACA<br>GAGATCTACCAGGCAGGCTCCACCCCTTGCAACGGAGTGGAG<br>GGCTTCAATTGTTATTTCCCCTGCAGAGCTACGGCTTCCAGCC<br>TACAAATGGCGTGGGCTATCAGCCATACAGGGTGGTGGTGCTG<br>TCCTTTGAGCTGCTGCACGCACCTGCAACCGTGGGGAGTTCCG<br>GTGGTGGCGGGAGCGGAGGTGGAGGCTCGAGCGGTGGATGGC<br>CATGGTACATTTGGCTAGGTTTTATAGCTGGCTTGATTGCCATA<br>GTAATGGTGACAATTATGCTTTGCTGTATGACCAGTTGCTGTAG<br>TTGTCTCAAGGGCTGTTGTTCTTGTGGTTCCTGCTGCAAATTTGA<br>TGAAGACGACTCTGAGCCAGTGCTCAAAGGAGTCAAATTACA<br>TTACACATATCCGTATGATGTTCCGGATTATGCTTAG	METDTLLLWVLLLWV<br>PGSTGDSGSSGSGSGD<br>YKDHDGDYKDHDID<br>YKDDDDKGGGGSSG<br>GNITNLCPFGEVFNA<br>TRFASVYAWNRKRIS<br>NCVADYSVLYNSASF<br>STFKCYGVSPTKLNDL<br>CFTNVYADSFVIRGDE<br>VRQIAPGQTGKIADY<br>NYKLPDDFTGCVIAW<br>NSNNLDSKVGGNYN<br>YLYRLFRKSNLKPFER<br>DISTEIYQAGSTPCNG<br>VEGFNCYFPLQSYGF<br>QPTNGVGYQPYRVVV<br>LSFELLHAPATVGSSG<br>GGGSGGGGSSGGWP<br>WYIWLGFIAGLIAIVM<br>VTIMLCCMTSCCSCL<br>KGCCSCGSCCKFDED<br>DSEPVLKGVKLHYTY<br>PYDVPDYA

© 2020 by the authors. Submitted for possible open access publication under the terms and conditions of the Creative Commons Attribution (CC BY) license (http://creativecommons.org/licenses/by/4.0/).
